# Cross-Domain Fault Diagnosis of Rotating Machinery Under Time-Varying Rotational Speed and Asymmetric Domain Label Condition

**DOI:** 10.3390/s25092818

**Published:** 2025-04-30

**Authors:** Siyuan Liu, Jinying Huang, Peiyu Han, Zhenfang Fan, Jiancheng Ma

**Affiliations:** 1 School of Data Science and Technology, North University of China, Taiyuan 030051, China; jyhuang@nuc.edu.cn; 2 School of Mechanical Engineering, North University of China, Taiyuan 030051, China; 20230200@nuc.edu.cn (Z.F.); jianchengma@126.com (J.M.); 3 College of Professional Studies, Northeastern University in Silicon Valley, San Jose, CA 95113, USA; han.peiy@northeastern.edu

**Keywords:** fault diagnosis, domain adaptation, time-varying speed, asymmetric label space

## Abstract

In practical engineering, the asymmetric problem of the domain label space is inevitable owing to the prior fault information of the target domain being difficult to completely obtain. This implies that the target domain may include unseen fault classes or lack certain fault classes found in the source domain. To maintain diagnostic performance and knowledge generalization across different speeds, cross-domain intelligent fault diagnosis (IFD) models are widely researched. However, the rigid requirement for consistent domain label spaces hinders the IFD model from identifying private fault patterns in the target domain. In practical engineering, the asymmetric domain label space problem is inevitable, as the target domain’s fault prior information is difficult to completely obtain. This means that the target domain may have unseen fault classes or lack some source domain fault classes. To address these challenges, we propose an asymmetric cross-domain IFD method with label position matching and boundary sparse learning (ASY-WLB). It reduces the IFD model’s dependence on domain label space symmetry during transient speed variation. To integrate signal prior knowledge for transferable feature representation, angular resampling is used to lessen the time-varying speed fluctuations’ impact on the IFD model. We design a label-positioning information compensation mechanism and weighted contrastive domain discrepancy, accurately matching unseen class label information and constraining the diagnosis model’s decision boundary from a data conditional distribution perspective. Finally, extensive experiments on two time-varying speed datasets demonstrate our method’s superiority.

## 1. Introduction

Transient changes in rotational speed are common in the operation of rotating machinery equipment. These changes include the start and stop of a piece of equipment triggered by a wide range of speed changes, dramatic speed fluctuations due to changes in load and operating conditions, etc. [1]. Under time-varying rotational speeds, the repetition frequency of transient impulses excited by a local fault will be altered, resulting in fault-related frequencies that exhibit “frequency ambiguous” non-discretization in the spectrum [2,3]. For this problem, common signal processing methods with physical interpretability, such as stochastic resonance [4], order tracking [5], and sparse representation [6], have been widely studied [7]. However, these methods rely on the expert’s rich experience in fault diagnosis and in-depth analysis of the fault mechanism of a specific monitoring object, making it challenging to realize intelligent and rapid fault diagnosis.

Benefiting from the advancement of next-generation artificial intelligence technology, deep-learning end-to-end IFD methods have received widespread attention in the industrial sector and have been applied to the fault diagnosis of rotating machinery under time-varying speed conditions [8,9]. Unfortunately, the non-interpretability of deep learning complicates the reliability of fault diagnosis results, limiting its acceptance in engineering applications [10]. Consequently, signal-processing-empowered deep learning has emerged as an important development trend in current IFD [11]. In recent studies, in order to enhance the performance and interpretability of deep-learning models, some scholars have combined physical a priori information on rotating machinery and advanced signal-processing methods to construct input samples for diagnostic model training, through which they emphasize the importance of model input features [12,13,14]. Therefore, combining traditional signal-processing methods to eliminate the interference of time-varying rotational speed on IFD will be highly significant. Otherwise, in practical engineering applications, we often need to complete diagnostic knowledge transfer from the laboratory to real industrial scenarios. However, the statistical distribution of the monitoring data is changed by fluctuations in the parameters, such as load and rotational speeds, which ultimately make it challenging to transfer fault knowledge from one operating condition (i.e., source domain) to another one (i.e., target domain) using deep-learning models. Therefore, the study of cross-domain IFD based on domain adaptation (DA) is considered a highly intriguing area of research. Unsupervised DA (UDA) techniques bridge domain discrepancies by transferring highly relevant knowledge from source domains with abundant labeled samples to target domains lacking labeled samples. This allows the extraction of domain-invariant features under different conditions [15].

UDA-based cross-domain IFD methods can be broadly categorized as discrepancy-based DA [16,17,18] and adversarial-based DA [19,20,21]. Discrepancy-based DA explicitly reduces the statistical distribution discrepancies between different data domains in one or more specific distance spaces by minimizing the distance function. In contrast, adversarial-based DA, by cheating the domain discriminator to implicitly align the source and target domains, lessens the interference caused by statistical distribution discrepancies on fault classifiers. Adversarial-based DA is obviously more adaptive than the former since it eliminates the need to account for the distance metric loss during training. Therefore, with reduced computational overhead, adversarial-based DA autonomously mitigates the interference in diagnostic models caused by transient changes in signal time scales. Li et al. reduce the distance of high-dimensional mapping features between different domains based on minimizing the max mean discrepancy (MMD) loss for knowledge transfer across devices in rotating machinery [22]. Jiao et al. recognize bearing faults and gear faults at different speeds and loads based on joint MMD and an adversarial learner [23]. Zhou et al. proposed a stochastic contrast regularization method inspired by contrast-learning theory, which encourages the model to learn domain-invariant features independent of working conditions and improves the model’s performance in working condition generalization [24]. Li et al. conducted extensive research on UDA-based IFD research in recent years from an industrial perspective [25]. To address the problem of the data without annotation under new working conditions in coupling IFD, Xiao et al. proposed a multi-label deep transfer-learning method [26]. To reduce the interference of time-varying rotational speed and weakly supervised data conditions, Liu et al. proposed a cross-domain IFD method based on complementary-label learning [27]. It is worth mentioning that IFD methods based on meta-learning [28] ideas have recently gained momentum, such as zero-shot learning [29] and one-shot learning [30]. Different from UDA, their training process does not depend much on the target data. However, the meta-learning approach requires obtaining the class description or the data attributes of the target domain, which makes it difficult to handle black box problems, such as unknown faults or unknown fault domains. In summary, most UDA-learning scenarios for cross-domain IFD methods often require that the source and target domains share the labeling space, i.e., closed-UDA (CUDA)-learning conditions.

**Figure 1:**
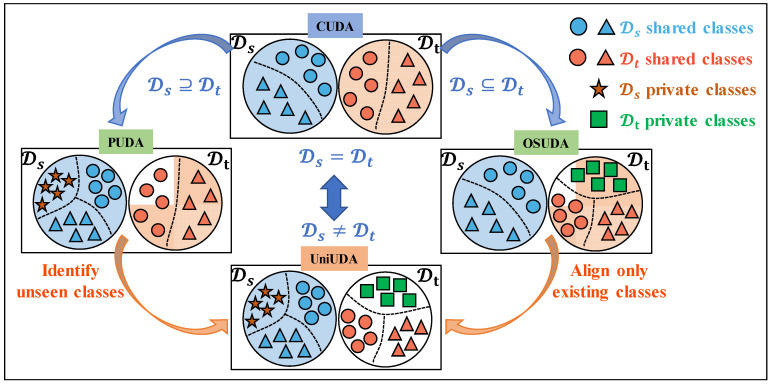
Illustrations of the different cross-domain IFD scenarios.

Considering the cost of acquiring and annotating industrial field data, the source domain is limited in terms of labeled categories, and the source and target domains are hardly guaranteed to be perfectly symmetric. Figure 1 shows the cross-domain IFD scenarios that may occur in real industries. When private fault modes that are unknown to the source domain occur in the target domain, the domain-invariant knowledge learned based on the source domain labeled data cannot meet the needs of cross-domain IFD. To address this challenge, Zhao et al. considered two domain label space asymmetry scenarios with private labels in the target domain and private labels in both the source and target domains to accomplish cross-domain IFD in the corresponding open-set UDA (OSUDA) and universal UDA (UniUDA) scenarios, respectively [31]. Zhang et al. successively combined adversarial learning in OSUDA and UniUDA learning scenarios and designed different weighting mechanisms to overcome the non-ideal data conditions of domain label space asymmetry [32,33]. The UDA method mentioned in the above research has a wide range of application prospects. However, a number of challenging issues remain in the current study. (i) The UDA problem under time-varying operating conditions has received limited attention in most UDA-technique research, despite the fact that fault-independent changes in operating conditions, such as speed and load, typically lie between the dynamic and the steady state. (ii) There is insufficient research on cross-domain IFD problems in UniUDA scenarios. UniUDA can be considered as a composite problem of partial UDA [34] (PUDA) and OSUDA [35]. In real industries, the source domain may contain private fault types that have not occurred in the target domain, and this knowledge may interfere with the knowledge transfer performance of OSUDA. (iii) For samples of unknown types in the target domain, the decision space is squeezed in the UniUDA problem setting. Specifically, when an unknown fault type exists in the target domain, the IFD model cannot learn representative features from the source domain information that distinguish known faults from unknown faults. Therefore, in the target domain, including knowledge of the unknown faults, the IFD model should maximize the distance between dissimilar samples and minimize the distance between similar samples. In this way, it can make the samples with ambiguous categorical attributes near the decision boundary in the sample space sparser, so that the domain-invariant features can be better learned. In order to solve the above problems, in the UniUDA scenario setting, we propose the ASY-WLB cross-domain IFD method based on the monitoring signal obtained at time-varying rotational speeds. Based on bearing data from the University of Ottawa [36] and vibration data under time-varying rotational speed conditions of planetary gearboxes in our laboratory, we validated the proposed method. In two separate cases, we set up a range of learning scenarios in different data domains with asymmetric label space to validate the effectiveness of the proposed method. The results indicate that the proposed method is capable of both efficiently identifying the private-type samples across various domains and aligning the shared-type samples within those domains. The innovations in this paper are summarized as follows:

(1) We construct the model’s input features with physical priors through angular resampling, which explicitly reduces the distribution drift of the signal on the time scale. This makes the IFD model interpretable during the process of learning the discriminative features independent of the rotational speed in the condition-monitoring data;
(2) We propose a label-positioning information compensation mechanism for the discriminative learning of shared and unknown classes in the source and target domains by sample-wise alignment learning and class-wise alignment learning. ASY-WLB uses a prediction margin index (PMI) to determine the probability that samples belong to shared classes, thereby increasing confidence in the correspondence between the samples from the target domain and pseudo-labels from the classifier outputs;
(3) We used balanced learning of intra-class compactness and inter-class separation. To effectively leverage the known sample-type versus the private sample-type potential label information, we proposed a weighted contrastive domain discrepancy (WCDD) loss that aims to minimize the distance between similar samples and maximize the distance between dissimilar samples implicitly, and embed this into the learning process of the ASY-WLB models.

The remainder of this paper is organized as follows. The proposed method is described in Section 2. Experiments are carried out for validation and analysis in Section 3. Conclusions are drawn in Section 4.

## 2. Methodology

### 2.1. Problem Formulation

In the UniUDA scenario setting, the sample space of the source domain $D_{s}={\left\{x_{i}^{s},y_{i}^{s}\right\}}_{i=1}^{n_{s}}$ contains $n_{s}$ labeled samples, and the sample space of the target domain $D_{t}={\left\{x_{i}^{t}\right\}}_{i=1}^{n_{t}}$ contains $n_{t}$ unlabeled samples. The number of classes in the source and target domains is $K_{s}$ and $K_{t}$. The samples in both $D_{s}$ and $D_{t}$ are from the envelope spectral signals that have been resampled in the angular domain under time-varying rotational speed conditions. The samples in the target domain may be affiliated with some of the known sample types in the source domain, or all of the known sample types, and may also include sample types that are not present in the source domain.

Let $C_{s}$ and $C_{t}$ represent the set of labels in the source and target domains, respectively, and let $C=C_{s}\cap C_{t}$ be the set of shared labels in both domains. $\bar{C_{s}}=C_{s}-C$ represents the set of private labels in the source domain, which are known during model training. $\bar{C_{t}}=C_{t}-C$ represents the set of private labels within the target domain that correspond to unknown fault modes, for which there is no prior knowledge in the training phase of the IFD model. The Jaccard distance $\xi =C/\left(C_{s}\cup C_{t}\right)$ is used to define a specific UDA learning scenario, where ξ can be any rational number between 0 and 1. When ξ=1, the learning scenario for cross-domain IFD degenerates into CUDA. The learning objective of the IFD model is to acquire domain-invariant knowledge from known information in the source domain and effectively apply it to the unlabeled samples in the target domain. In other words, the IFD model will work stably with different ξ, even if ξ is unknown. While excluding the interference of private fault types in the source domain, the IFD model accurately classifies shared sample types C in the target domain and identifies private unknown fault types ${\bar{C}}_{t}$ in the target domain.

### 2.2. Representation of Rotational Speed-Independent Transferable Features

Variations in parameters like load, rotational speed, and others may cause potential discrepancies in the statistical distribution of the monitoring data across time scales in cross-domain IFD scenarios. Time-varying rotational speeds cause the instantaneous frequencies of key signal components, including fault components, intrinsic components, and their harmonics, to change on different time scales. This problem will further amplify the intra-class distance with a continuous domain distribution shift, causing concept drift within the monitored data class ($P_{s}\left(x_{i}^{s}\right)\neq P_{s}\left(x_{i+1}^{s}\right)$) and conditional shift between the data domains ($P_{s}\left(x\left|y\right.\right)\neq P_{t}\left(x\left|y\right.\right)$) [37]. This makes it difficult for IFD models to intuitively capture periodic features in the training samples, resulting in significant performance deterioration during the fault prediction phase.

Therefore, we use signal-processing methods to reduce the interference of time-varying rotational speeds in the raw data, which can further improve the performance and generalization ability of the ASY-WLB model. By combining signal-processing techniques, the ASY-WLB model can balance its interpretability and intelligence. Velocity-dependent frequencies can be transformed into relatively stable orders via angular resampling, which allows the overlapping single components of the time-domain signal to be separated in the angle-domain signal’s spectrum [38]. Resampling the data in multiple domains reduces the interference of transient rotational speed fluctuations on the learning process of the ASY-WLB model, hence increasing the model’s robustness. Meanwhile, combining the physical prior information of rotating machinery to complete the angular resampling also enhances the interpretability of the input features of the ASY-WLB model. The steps are as follows:

Step 1: Obtain the vibration signal $x=\left\{x\left(1\right),\cdots ,x\left(N\right)\right\}$ and its physical parameters for the monitored equipment under the present operating conditions, where N is the number of signal sampling points. The instantaneous rotational frequency $f_{r}$ and the physical parameters of the item under observation are used to compute an important instantaneous frequency $f_{e}$. For each set f of instantaneous frequencies to be concerned with, depending on the number of samples $R_{s}$ per unit rotation, the Nyquist frequency [39] can be expressed as:

(1) $$f_{Nyquist}=\frac{R_{s}min\left(f\right)}{2}$$

Step 2: The pulse time node, f, indicates the angle at which the reference axis has been turned, and interpolation is used to determine the time stamp for sampling at equal angular intervals. The vibration signal is interpolated according to the time stamp to transform the time domain information into angle domain information;

Step 3: The envelope spectrum of the signal in the angle domain is computed using the Hilbert transform. The obtained envelope spectrum signal retains the fault-related, but rotational speed-independent, feature components, which will be used in subsequent experiments to learn the IFD model.

### 2.3. Cross-Domain IFD Method Based on ASY-WLB

#### 2.3.1. IFD Method Across Asymmetric Domain Based on UniUDAN

**Figure 2:**
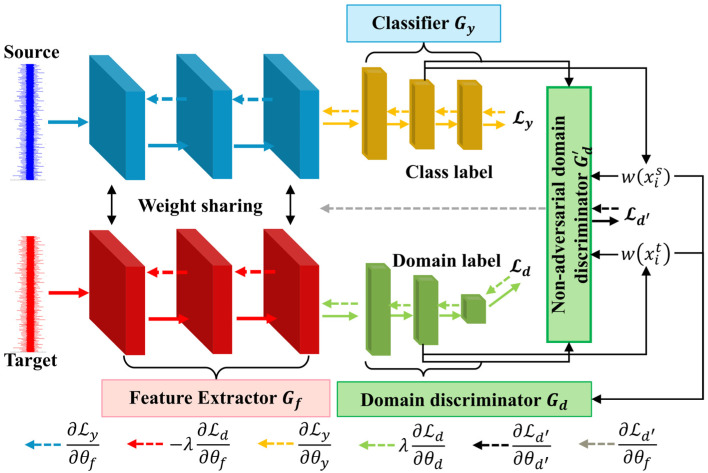
Schematic diagram of UniUDAN.

For the problem of asymmetric label space between the source and target domains, the fault diagnosis model based on the UniUDA network (UniUDAN) is shown in Figure 2. The UniUDAN model structure consists of a feature extractor $G_{f}$, a classifier $G_{y}$, a domain discriminator $G_{d}$, and a non-domain discriminator $G_{d^{\prime }}$. Let $\theta_{f}$, $\theta_{y}$, $\theta_{d}$, and $\theta_{d^{\prime }}$ denote the parameters of $G_{f}$, $G_{y}$, $G_{d}$, and $G_{d^{\prime }}$, respectively. The loss function of UniUDAN includes cross-entropy classification loss $L_{y}$, weighted domain discriminator loss $L_{d}$, and non-adversarial domain discriminator loss $L_{d^{\prime }}$. $G_{d}$ is used to enforce domain alignment for both the source and target domains using the set of labels C that are common between them, thus matching their respective feature distributions. It achieves domain confusion by fooling the domain discriminator $G_{d}$ and mistakenly labeling samples from the target domain as belonging to the source domain. $G_{d^{\prime }}$ is used to quantify the degree of similarity between the current sample and the different data domains, tending to 1 when it belongs to the source domain and 0 when it belongs to the target domain. The domain similarity $d^{\prime }$ can be expressed as:

(2) $$d^{\prime }=G_{d^{\prime }}(G_{f}(x))$$

The uncertainty of the prediction results is measured in terms of information entropy. We assume that:

(3) $$E_{x∼q_{{\bar{C}}_{t}}}H\left(p\right)>E_{x∼q_{C}}H\left(p\right)>E_{x∼p_{C}}H\left(p\right)>E_{x∼p_{{\bar{C}}_{s}}}H\left(p\right)$$

where p and q denote the distributions of the source and target domains, respectively. The subscripts denote the set of types of affiliation. $H\left(\cdot \right)$ denotes the result of the information entropy calculation. $L_{y}$, $L_{d}$, and $L_{d^{\prime }}$ can be represented as follows:

(4) $$L_{y}\left(\theta_{f},\theta_{y}\right)=-E_{\left(x_{i}^{s},y_{i}^{s}\right)\in D_{s}}\sum_{c=0}^{K_{s}-1}I\left[y_{i}^{s}=c\right]\times \log\left[G_{y}\left(G_{f}\left(x_{i}^{s};\theta_{f}\right);\theta_{y}\right)\right]$$

(5) $$L_{d\;}\left(\theta_{f},\theta_{d}\right)=-E_{x_{i}^{s}\in D_{s}}\omega \left(x_{i}^{s}\right)\log\left[G_{d}\left(G_{f}\left(x_{i}^{s};\theta_{f}\right);\theta_{d}\right)\right]-E_{x_{i}^{t}\in D_{t}}\omega \left(x_{i}^{t}\right)\log\left[1-G_{d}\left(G_{f}\left(x_{i}^{t};\theta_{f}\right);\theta_{d}\right)\right]$$

(6) $$L_{d^{\prime }}\left(\theta_{f},\theta_{d^{\prime }}\right)=-E_{x_{i}^{s}\in D_{s}}\log\left[G_{d^{\prime }}\left(G_{f}\left(x_{i}^{s};\theta_{f}\right);\theta_{d\prime }\right)\right]-E_{x_{i}^{t}\in D_{t}}\log\left[1-G_{d^{\prime }}\left(G_{f}\left(x_{i}^{t};\theta_{f}\right);\theta_{d\prime }\right)\right]$$

where $I\left[\cdot \right]$ is the indicator function. $\omega \left(x_{i}^{s}\right)$ and $\omega \left(x_{i}^{t}\right)$ represent the probabilities that the category labels for the source domain sample $x_{i}^{s}$ and the target domain sample $x_{i}^{t}$, respectively, are included in the shared label set C. We use domain similarity and information entropy to quantify the instance-level transferability criterion, assigning higher weights to samples with lower uncertainties. The sample weight coefficients for the source and target domains are denoted, respectively:

(7) $$\omega^{S}\left(x_{i}^{s}\right)=\frac{H\left(p\left(x_{i}^{s}\right)\right)}{\log\left|C_{s}\right|}-d^{\prime }\left(x_{i}^{s}\right)$$

(8) $$\omega^{S}\left(x_{i}^{t}\right)=d^{\prime }\left(x_{i}^{t}\right)-\frac{H\left(p\left(x_{i}^{t}\right)\right)}{\log\left|C_{s}\right|}$$

$\log\left|C_{s}\right|$ is the maximum value of the entropy of the classification result, allowing $H\left(p\left(\cdot \right)\right)$ to be normalized to the interval $\left[0,1\right]$. Here, we predefine a threshold $\omega_{0}$, with the aim of making $d^{\prime }\left(x_{i}^{t}\right)$ as large as possible and $H\left(p\left(x_{i}^{t}\right)\right)$ as small as possible. When $\omega \left(x_{i}^{t}\right)<\omega_{0}$, we then consider $x_{i}^{t}$ to be a sample of unknown type. Ultimately, the parameter optimization objective of UniUDAN can be represented as follows:

(9) $$\left({\hat{\theta }}_{f},{\hat{\theta }}_{y}\right)=\underset{\theta_{f},\theta y}{\arg\min}L_{y}\left(\theta_{f},\theta_{y}\right)-\lambda_{d}L_{d}\left(\theta_{f},{\hat{\theta }}_{d}\right)$$

(10) $$\left({\hat{\theta }}_{d}\right)=\underset{\theta_{d}}{\arg\min}L_{d}\left({\hat{\theta }}_{f},{\hat{\theta }}_{y},\theta_{d}\right)$$

(11) $$\left({\hat{\theta }}_{d^{\prime }}\right)=\underset{\theta_{d^{\prime }}}{\arg\min}L_{d}\left({\hat{\theta }}_{f},\theta_{d^{\prime }}\right)$$

where $\left({\hat{\theta }}_{f},{\hat{\theta }}_{y},{\hat{\theta }}_{d},{\hat{\theta }}_{d^{\prime }}\right)$ is the optimal parameter at the saddle point, and $\lambda_{d}$ is the penalty coefficient of $G_{d}$.

#### 2.3.2. Label-Positioning Information Compensation Mechanism

**Figure 3:**
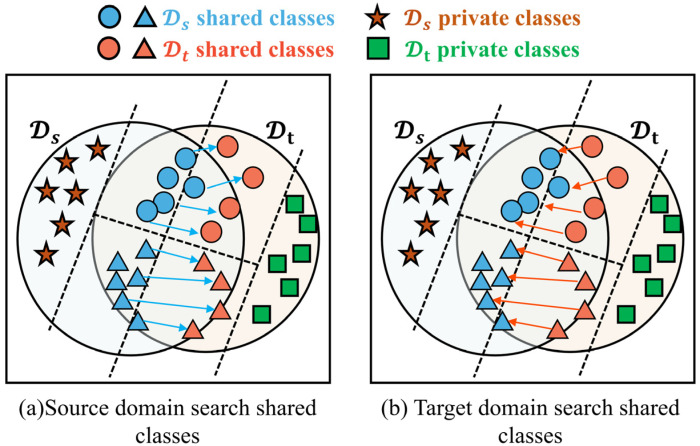
Feature alignment mechanism for feature extractors $G_{f}$.

According to the transferability criterion of UniUDAN, it is assumed that the prediction entropy of the samples from $D_{s}$ becomes larger because they are influenced by the high entropy samples from $D_{t}$ [35]. As shown in Figure 3a, we prefer that $D_{t}$ determines the shared versus private classes in $D_{s}$, i.e., prioritizes the shared class samples in $D_{s}$ to force alignment to the shared class samples in $D_{t}$. However, as shown in Figure 3b, $G_{f}$ prefers to move the target distribution to the source ones in the UniUDAN adversarial-learning process, since this is the more-efficient way to simultaneously minimize classification loss and maximize the domain loss. This results in a smaller prediction entropy of the samples from $D_{s}$, rather than a larger prediction entropy of samples from $D_{t}$. Obviously, UniUDAN is unable to capture the discriminative information of shared versus private classes from the perspective of class-labeling information.

Therefore, we propose a label-positioning information compensation mechanism that enhances ASY-WLB perception of transferable features, so that $D_{s}$ and $D_{t}$ can realize both sample-wise alignment and class-wise alignment. First, we measure the sample-wise alignment by retaining the non-adversarial domain discriminator $G_{d^{\prime }}$ and, thus, utilizing the instance-level transferability criterion, corresponding to $\omega^{S}\left(x_{i}^{s}\right)$ and $\omega^{S}\left(x_{i}^{t}\right)$ in Equations (7) and (8). Next, we utilize the prediction margin of the target samples [40] to measure the degree of class-wise alignment. The PMI is defined as the maximum prediction probability of the classifier $G_{y}$ minus the second largest prediction probability. Based on the PMI, the IFD model can measure the pseudo-labeling confidence of the target domain samples. The pseudo-labeling of the target domain samples is determined based on the a priori information in the source domain. The PMI of classifier $G_{y}$ at a pseudo-labeled sample x can be defined as:

(12) $$m_{y}(x)≜G_{y}(x,\hat{y})-\max_{y\neq \hat{y}}G_{y}\left(x,y\right)$$

(13) $$\hat{y}≜\arg\underset{y}{\max}G_{y}\left(x,y\right)$$

Then, the PMI vector of the data distribution D can be expressed as:

(14) $$M(D,G_{y})≜E_{x\in D}{\left[m_{y}(\{x|\hat{y}=1\}),\cdots ,m_{y}(\{x|\hat{y}=n_{y}\})\right]}^{T}$$

To ensure that the PMIs of $D_{t}$ are updated along with the IFD model, we record the updating process of M through a $C_{s}$-dimensional PMI space $V_{M}$. The update process is as follows:

(15) $$V_{M}^{t+1}=\frac{1}{t+1}(t\times V_{M}^{t}+M(D_{t},G_{y}))$$

where $t\times V_{M}^{t}$ is the accumulated PMI over the previous t steps. And t<T, where T is the maximum step set before training. Each $V_{M}$ denotes the empirical PMI by which the predictions of unlabeled samples in $D_{t}$ belong to the source domain class. To ensure class-wise alignment, the class-weight coefficients for the source and target domains are denoted, respectively:

(16) $$w^{C}(x_{i}^{s})=\frac{V_{M}[y_{i}^{s}]-\underset{x\in D_{s}}{\min}V_{M}[y_{i}^{s}]}{\underset{x\in D_{s}}{\max}V_{M}[y_{i}^{s}]-\underset{x\in D_{s}}{\min}V_{M}[y_{i}^{s}]}$$

(17) $$w^{C}(x_{i}^{t})=\frac{\underset{c\in C_{s}}{\max}G_{y}^{c}\left(G_{f}(x_{i}^{t})\right)-\underset{x\in D_{t}}{\min}\left[\underset{c\in C_{s}}{\max}G_{y}^{c}\left(G_{f}(x_{i}^{t})\right)\right]}{\underset{x\in D_{t}}{\max}\left[\underset{c\in C_{s}}{\max}G_{y}^{c}\left(G_{f}(x_{i}^{t})\right)\right]-\underset{x\in D_{t}}{\min}\left[\underset{c\in C_{s}}{\max}G_{y}^{c}\left(G_{f}(x_{i}^{t})\right)\right]}$$

Ultimately, we denote the probability that samples from $D_{s}$ and samples from $D_{t}$ belong to the set of shared labels as:

(18) $$\left\{\begin{array}{l} w(x_{i}^{s})=\left(w^{S}(x_{i}^{s})+w^{C}(x_{i}^{s})\right)/2\\ w(x_{i}^{t})=\left(w^{S}(x_{i}^{t})+w^{C}(x_{i}^{t})\right)/2 \end{array}\right.$$

In summary, we adopt a probabilistic approach to implement a label-positioning information compensation mechanism in order to incorporate the prediction margin loss of $G_{y}$ into UniUDA scenarios. Based on the sample-wise alignment learning of the non-adversarial domain discriminator $G_{d^{\prime }}$, we further realize the class-wise alignment learning by weighting the PMI of $D_{t}$ corresponding to $D_{s}$. Meanwhile, class-wise alignment enhances the confidence of the IFD model’s prediction results, ensuring that $G_{y}$ annotates the known pseudo-labels with higher confidence for unlabeled samples from $D_{t}$.

#### 2.3.3. Balanced Learning of Intra-Class Compactness and Inter-Class Separation

The UniUDAN model adjusts the weights of the shared-labeled samples and private-labeled samples by assessing the consistency of the statistical distributions of the samples to be predicted in the target domain and those in the source domain. This eliminates all interference caused by private classes on the learning of shared classes. The adversarial domain discriminator $G_{d}$ can learn domain-invariant features from the samples of shared classes, reducing the distributional difference between the source and target domains implicitly. However, the sample space may contain many samples that are ambiguous in terms of class when no constraints are placed on the sample space. These ambiguous samples are typically found around the decision boundary between the unknown and known classes, which can easily lead to a misalignment of the distribution of unknown and known fault classes in the feature space.

To address the aforementioned issue, we have referred to the article [41,42] and proposed the ASY-WLB based on WCDD loss to improve UniUDAN. In order to reduce the intra-class distance and maximize the inter-class distance in the sample feature-mapping space, we incorporate the WCDD optimization objective into the domain adversarial-learning process. Eventually, samples of the same class from the source and target domains will have closer feature distances, heterogeneous samples will have farther feature distances, and samples near the class decision boundary will be sparser.

**Figure 4:**
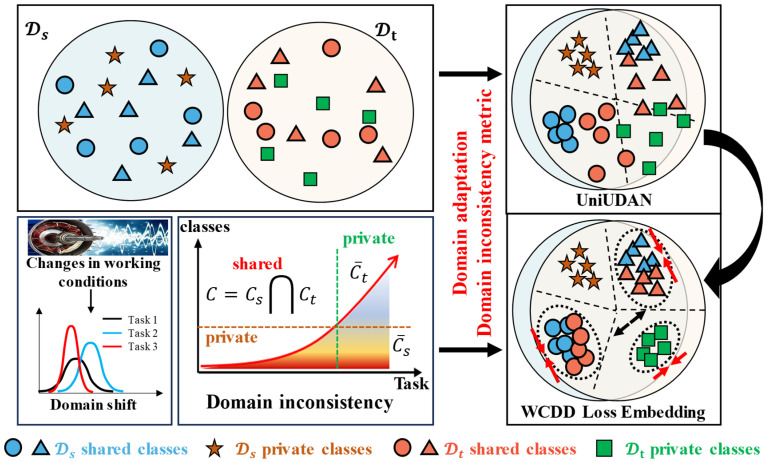
Illustration of weighted contrastive domain discrepancy loss embedding.

As shown in Figure 4, the ASY-WLB model embeds WCDD loss based on UniUDAN, and the improvement steps are as follows:

(1) First, ASY-WLB utilizes $G_{f}$ and $G_{y}$ to output the conditional distribution $P\left(G_{y}\left(G_{f}\left(X_{s}\right)\right)\right)$ (denoted as $P_{s}$) in $D_{s}$ and the conditional distribution $P\left(G_{y}\left(G_{f}\left(X_{t}\right)\right)\right)$ (denoted as $P_{t}$) in $D_{t}$, where $X_{s}={\left\{x_{i}^{s}\right\}}_{i=1}^{n_{s}}$ and $X_{t}={\left\{x_{i}^{t}\right\}}_{i=1}^{n_{t}}$;
(2) Next, the distribution discrepancy between $P_{s}$ and $P_{t}$ is calculated using the reproducing kernel Hilbert space (RKHS) embedding, denoted by $D\left(P_{s},P_{t}\right)$. The binary discriminant functions, corresponding to the two classes $c_{1}$ and $c_{2}$, are assumed:
  (19) $$\mu_{cc\prime }(y,y\prime )=\left\{\begin{array}{l} 1\;\;\;\;\;\mathrm{if}\;y=c,y\prime =c\prime \\ 0\;\;\;\;\;\mathrm{otherwise} \end{array}\right.$$
  where $c_{1}=c_{2}$ or $c_{1}\neq c_{2}$;
(3) Let ${\hat{f}}_{i}^{s}=G_{f}\left(x_{i}^{s}\right)$, ${\hat{f}}_{i}^{t}=G_{f}\left(x_{i}^{t}\right)$, ${\hat{y}}_{i}^{t}=G_{y}\left(G_{f}\left(x_{i}^{t}\right)\right)$ and ${\hat{y}}_{1:n_{t}}^{t}=\left\{{\hat{y}}_{1}^{t},{\hat{y}}_{2}^{t},\cdots ,{\hat{y}}_{n_{t}}^{t}\right\}$. Afterwards, the Gaussian kernel function $k\left(\cdot \right)$ is chosen to estimate the kernel mean embedding of the square term of $D(P_{s},P_{t})$, denoted by ${\hat{D}}^{c1c2}(\theta_{f},\theta_{y})$. ${\hat{D}}^{c1c2}(\theta_{f},\theta_{y})$ can be computed as:

(20) $${\hat{D}}^{c1c2}({\hat{y}}_{1:n_{t}}^{t},\theta_{f},\theta_{y})=e_{1}+e_{2}-2e_{3}$$

The sample-wise and class-wise transferability criterion weights of Equation (18) are integrated into the Equation (20), where $e_{1}$, $e_{2}$ and $e_{3}$ are, respectively, expressed as:

(21) $$e_{1}=\sum_{i=1}^{n_{s}}\sum_{j=1}^{n_{s}}\frac{\mu_{c_{1}c_{1}}\omega \left(x_{i}^{s}\right)\omega \left(x_{j}^{s}\right)\left(y_{i}^{s},y_{j}^{s}\right)k\left({\hat{f}}_{i}^{s},{\hat{f}}_{j}^{s}\right)}{\sum_{i=1}^{n_{s}}\sum_{j=1}^{n_{s}}\mu_{c_{1}c_{1}}\left(y_{i}^{s},y_{j}^{s}\right)}$$

(22) $$e_{2}=\sum_{i=1}^{n_{t}}\sum_{j=1}^{n_{t}}\frac{\mu_{c_{2}c_{2}}\omega \left(x_{i}^{t}\right)\omega \left(x_{j}^{t}\right)\left({\hat{y}}_{i}^{t},{\hat{y}}_{j}^{t}\right)k\left({\hat{f}}_{i}^{t},{\hat{f}}_{j}^{t}\right)}{\sum_{i=1}^{n_{t}}\sum_{j=1}^{n_{t}}\mu_{c_{2}c_{2}}\left({\hat{y}}_{i}^{t},{\hat{y}}_{j}^{t}\right)}$$

(23) $$e_{3}=\sum_{i=1}^{n_{s}}\sum_{j=1}^{n_{t}}\frac{\mu_{c_{1}c_{2}}\omega \left(x_{i}^{s}\right)\omega \left(x_{j}^{t}\right)\left(y_{i}^{s},{\hat{y}}_{j}^{t}\right)k\left({\hat{f}}_{i}^{s},{\hat{f}}_{j}^{t}\right)}{\sum_{i=1}^{n_{s}}\sum_{j=1}^{n_{t}}\mu_{c_{1}c_{2}}\left(y_{i}^{s},{\hat{y}}_{j}^{t}\right)}$$

when $c_{1}=c_{2}$, Equation (20) is used for intra-class compactness learning. When $c_{1}\neq c_{2}$, Equation (20) is used for inter-class separation learning. Ultimately, WCDD loss can be represented as:

(24) $$L_{wcdd}=\frac{1}{\left|K_{s}\right|}\sum_{c=1}^{\left|K_{s}\right|}{\hat{D}}^{cc}\left({\hat{y}}_{1:n_{t}}^{t},\theta_{f},\theta_{y}\right)-\frac{1}{\left|K_{s}\right|\left(\left|K_{s}\right|-1\right)}\sum_{c=1}^{\left|K_{s}\right|}\sum_{\begin{array}{c} c\prime =1\\ c\prime \neq c \end{array}}^{\left|K_{s}\right|}{\hat{D}}^{cc\prime }\left({\hat{y}}_{1:n_{t}}^{t},\theta_{f},\theta_{y}\right)$$

The first term of Equation (24) measures intra-class distance, and the second term measures inter-class distance. They will be optimized in the opposite direction. After embedding the WCDD loss and the label-positioning information compensation mechanism in UniUDAN, the optimization objective of the first term in Equation (9) is improved in ASY-WLB:

(25) $$\left({\hat{\theta }}_{f},{\hat{\theta }}_{y}\right)=\underset{\theta_{f},\theta y}{\arg\min}L_{y}\left(\theta_{f},\theta_{y}\right)-\lambda_{d}L_{d}\left(\theta_{f},{\hat{\theta }}_{d}\right)+\lambda_{wcdd}L_{wcdd}\left(\theta_{f},\theta_{y}\right)$$

#### 2.3.4. Model Structure and Optimization

**Figure 5:**
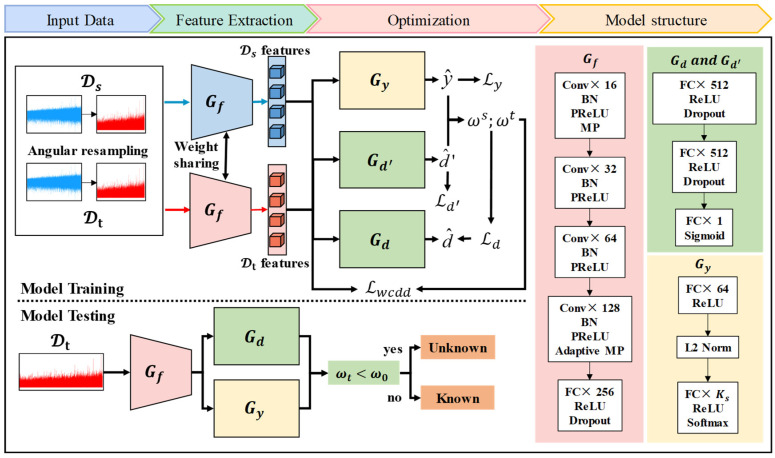
ASY-WLB cross-domain IFD model under time-varying rotational speed conditions.

The specific implementation process of ASY-WLB is illustrated in Figure 5. The feature extractor $G_{f}$ is structured with a convolutional layer (Conv), batch normalization (BN), max pooling (MP), adaptive max pooling (Adapt MP), a fully connected layer (FC), and a dropout. The activation function adopts the parametric rectified linear unit (PReLU). The adversarial domain discriminator $G_{d}$ utilizes the same linear structure as the non-adversarial domain discriminator $G_{d^{\prime }}$ and generates the probabilities of domain discrimination through a Sigmoid activation function layer. The classifier $G_{y}$ contains an L2-normalization (L2 Norm) layer and an activation function rectified linear unit (ReLU). The L2 Norm layer allows the network to bring the features of samples from the same class closer to each other during training, while amplifying the feature distances of samples from different classes. This enhancement improves the network’s classification confidence. The first module of the classifier output vector $G_{y}{\left(x\right)}_{1}$ becomes $\alpha G_{y}{\left(x\right)}_{1}/\left\|G_{y}{\left(x\right)}_{1}\right\|$ after the L2 Norm. α represents the shrinkage coefficient. We use the stochastic gradient descent (SGD) algorithm for parameter optimization of the proposed model. The SGD optimizer can better inhibit fluctuations in the learning rate during the later training stage to obtain an optimal solution [43]. The optimization process for the parameters $\theta_{f}$, $\theta_{y}$, $\theta_{d}$, and $\theta_{d^{\prime }}$ of the different ASY-WLB components is as follows:

(26) $$\theta_{f}\leftarrow \theta_{f}-\varepsilon \left(\frac{\delta L_{y}}{\delta \theta_{f}}+\lambda_{cdd}\frac{\delta L_{cdd}}{\delta \theta_{f}}+\lambda_{d}\frac{\delta L_{d}}{\delta \theta_{f}}+\frac{\delta L_{d\prime }}{\delta \theta_{f}}\right)$$

(27) $$\theta_{y}\leftarrow \theta_{y}-\varepsilon \left(\frac{\delta L_{y}}{\delta \theta_{y}}+\lambda_{cdd}\frac{\delta L_{cdd}}{\delta \theta_{y}}+\lambda_{d}\frac{\delta L_{d}}{\delta \theta_{y}}\right)$$

(28) $$\theta_{d}\leftarrow \theta_{d}-\varepsilon \left(\lambda_{d}\frac{\delta L_{d}}{\delta \theta_{d}}\right)$$

(29) $$\theta_{d^{\prime }}\leftarrow \theta_{d^{\prime }}-\varepsilon \left(\frac{\delta L_{d\prime }}{\delta \theta_{d\prime }}\right)$$

where ε denotes the learning rate of the optimizer, and $\delta \left(\cdot \right)$ denotes the partial derivative algorithm.

## 3. Experimental Verification

### 3.1. Case Description

In this section, we formulate 16 cross-domain IFD tasks, where the domain label spaces are asymmetric, to evaluate the performance of the ASY-WLB method on two datasets with time-varying rotational speed fluctuations. Meanwhile, we employ the following common baselines to demonstrate the effectiveness and superiority of the proposed ASY-WLB method in case studies to facilitate a comprehensive evaluation. Specifically, in order to minimize the interference of speed trends with the experimental results, for different DA scenarios, we choose data samples with similar speed trends under different load conditions as much as possible to validate our approach. We also designed a small number of DA tasks with different speed trends in the two cases to prove the robustness of ASY-WLB to rotational speed fluctuations. All data from two case studies were feature-transformed according to the method in Section 2.2 and overlapped sampled. The sample length of the unit data was uniformly set to 5000 for both cases. Combined with the actual engineering, long-term monitoring often produces more health data than fault data and exhibits class-imbalance problems. Thus, in two cases, the number of our health samples is approximately twice the number of samples for each fault type. The number of training and test datasets will change depending on the DA scenarios.

#### 3.1.1. Case Study 1

**Figure 6:**
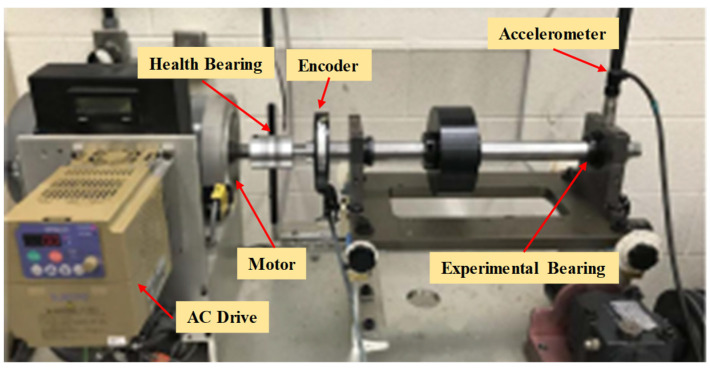
Experimental set-up in case 1.

In case study 1, we utilized the ER16K bearing fault datasets sourced from the University of Ottawa, shown in Figure 6 [36]. The test rig comprises six key components: an AC drive, a motor, an incremental encoder (EPC model 775), an accelerometer (ICP accelerometer, Model 623C01), an experimental bearing, and a health bearing. The shaft is driven by a motor, and the rotational speed is controlled by an AC drive. An encoder is used to record shaft rotational speeds. The sampling frequency of all data is 200 kHz. The monitored samples are classified as healthy (H), inner-ring fault (I), outer-ring fault (O), rolling element fault (B), and compound fault (C). Each class contains four different speed-varying conditions: increasing speed (IS), decreasing speed (DS), increasing then decreasing speed (IS then DS), and decreasing then increasing speed (DS then IS), corresponding to the label annotations A, B, C, and D. According to the parameters of the bearings, the ball-pass frequency of the inner race (BPFI) is equal to 5.43 times the shaft rotational frequency $F_{r}$, and the ball-pass frequency of the outer race (BPFO) is equal to 3.57 times the shaft rotational frequency $F_{r}$.

In addition, each speed-varying condition contained three different types of data, and the range of the speed fluctuations was inconsistent for each data type. Obviously, the difference in rotational speed trends from A to B is the largest, and therefore, the data has the largest degree of domain shift. The three data points for each speed-varying condition were grouped together in the experiment (e.g., OA contains O-A-1, O-A-2, and O-A-3). According to the UniUDA scenario setup, all the DA tasks are marked as O1-O8, respectively, and the specific information is shown in Table 1. Task O1 belongs to the CUDA scenario and can be considered as a performance upper boundary for the UniUDA scenario in case 1. O2 and O3 correspond to the cross-domain IFD scenarios for PUDA and OSUDA, respectively, and the same setup will be included in case study 2. In tasks O6 and O7, the shaft speed trends of the target domain samples are nonmonotonic. In task O8, the shaft speed trends of the samples in both the source and target domains are nonmonotonic.

#### 3.1.2. Case Study 2

**Figure 7:**
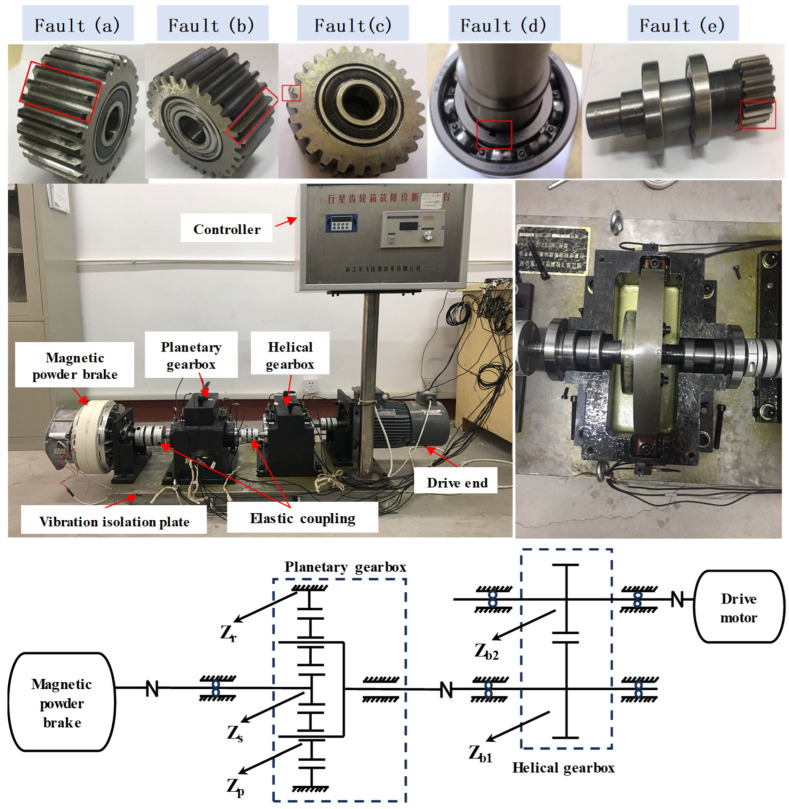
HFXZ-I planetary gearbox test rig. (**a**) Gear pitting. (**b**) Gear wear. (**c**) Gear crack. (**d**) Inner race defect. (**e**) Sun gear wear + (**a**).

In this case, the HFXZ-I planetary gearbox fault simulation test rig is shown in Figure 7. We installed acceleration sensors in the planetary gearbox, helical gearbox, and other key parts, respectively, and selected five channels of monitored data for experimental verification. The type of accelerometer used is CA-YD*. Three-directional vibration measurement points were set up at the center of the planetary gearbox’s top cover and base. Additionally, nine single-directional vibration measurement points were arranged at key locations and the input/output ends of the helical gearbox and planetary gearbox. The shaft speed sensor is mounted in a non-contact form on a second elastic coupling. The shaft rotational speed sensor is installed on the second elastic coupling in a non-contact form. The controller can adjust different shaft speeds and different load conditions as needed. The optional range of the load is 0–3 A, and the optional range of the shaft speed is 0–50 Hz.

Except for the healthy mode (H), we preconfigured five distinct fault modes in our experiments, including the compound fault of the sun gear wear and the planetary gear pitting (C), inner race of the bearing defect (I), planetary gear wear (W), planetary gear crack (K), and planetary gear pitting (P), corresponding to the labels of the training data from zero to five. We selected two load conditions corresponding to the load currents of 0.5A (denoted as A) and 1A (denoted as B), respectively. And we selected two speed conditions, namely increasing speed (IS, 0–50 Hz) and decreasing speed (DS, 50–0 Hz), corresponding to marker 1 and marker 2. According to the UniUDA scenario setup, all of the DA tasks are marked as T1–T8, respectively, and the specific information is shown in Table 2. For example, WA1 means that the class of this data is planetary wheel wear, the load is 0.5A, and the variation range of shaft speed is 0–50 Hz. Task O1 belongs to the CUDA scenario, and T2 and T3 correspond to the cross-domain IFD scenarios for PUDA and OSUDA. Tasks T3–T8 are distinguished by the fact that their source or target domains contain different classes, as well as quantitative differences. Compared to case study 1, the amount of information contained in the single sample for case study 2 is increased due to the increase in the number of sensor channels. Meanwhile, the range of shaft rotational speed in case study 2 is larger than that of the former, and the data for case study 2 also contains a small number of samples from the constant-speed condition.

### 3.2. Comparison Methods and Implementation Details

To comprehensively evaluate the performance of ASY-WLB, we present extensive results under four different settings: CUDA, PUDA, OSUDA, and UniUDA. We have no prior knowledge of the class information in $D_{t}$. We focused on the previous case study [33] and realized several more advanced IFD methods, demonstrating the superiority of the proposed method. Meanwhile, we also selected the classical model without DA capabilities for comparative experiments to ensure that our evaluation of ASY-WLB is more accurate and comprehensive. Except for the proposed methods (marked as M1), we compare our method with the previous state-of-the-art methods: (M2) the base model, (M3) DANN [19], (M4) OSBP [44], (M5) UniUDAN [35], and (M6) DCC [42]. Compared to M1, only $G_{f}$ and $G_{y}$ are retained in the model structure of M2. A domain adversarial neural network (DANN), which can reduce domain differences based on a domain adversarial learning strategy, focuses on solving CUDA problems. DANN embeds the domain discriminator $G_{d}$ in the structure of M2. Open-set DA by backpropagation (OSBP) detects and recognizes unknown classes in $D_{t}$. OSBP is modified from DANN by adding the neurons at the output that determine the unknown classes and can recognize private fault modes in $D_{t}$. UniUDAN does not carry out sparse learning near the class decision boundary, which will cause the feature distribution of the target domain to be more dispersed. Domain consensus clustering (DCC) aims to cluster both shared and unknown classes in order to better extract information from the latent space. However, DCC only focuses on sample-wise alignment learning without considering the class probability information, which can lead to overfitting of the source domain information.

For comparison, all the methods are composed using the underlying network architecture given in Figure 5, and the other training settings are determined by the related studies. The epochs of all methods are 300. The SGD algorithm can be selected as the optimization function. The model’s initial learning rate, momentum, and weight decay are set to 0.03, 0.9, and 1 × 10^−5^, respectively. The decay rate is 0.1, and the decay process occurs at 50 and 150 epochs. Throughout the experiments, $\omega_{0}=0.5$, and the trade-off parameters of $\lambda_{wcdd}$ and $\lambda_{d}$ in the proposed ASY-WLB are both set to 1. The shrinkage coefficient α is taken by two. The first convolutional layer has the same number of input channels as the number of sensor channels. These methods were tested on an Intel i9-13900K CPU, a GeForce RTX 4090 GPU, and Pytorch 2.0x.

### 3.3. Transferable Feature Extraction Based on Angular Resampling

**Figure 8:**
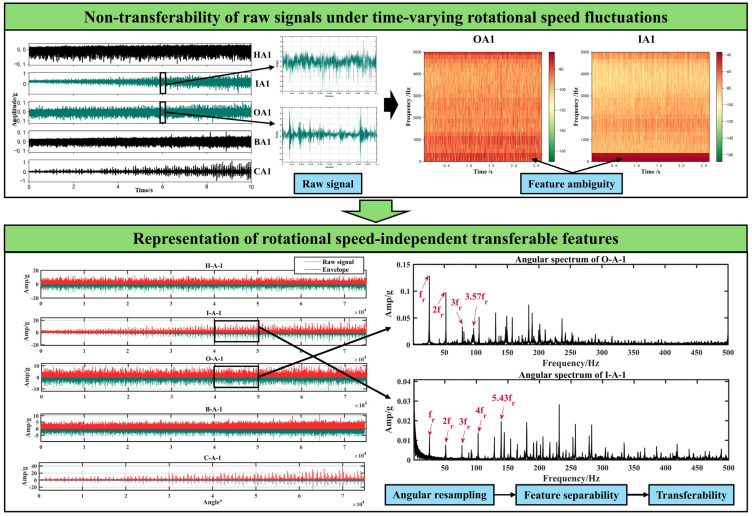
Extraction of transferable features.

For validation of the methodology, angular domain resampling was performed using the bearing data from the University of Ottawa, following steps (1) through (3) in Section 2.2. The process of extracting the transferable features is shown in Figure 8. Feature ambiguity implies inconsistency of data samples on time scales, which can lead to intra-class distribution shift. Obviously, the amplitude difference in the time domain is reduced in the envelope signal extracted from the angular domain compared to the original signal, which increases the intra-class similarity of the samples to some extent. Additionally, discretizing the signal frequency features will simplify the IFD model feature-decoupling process, thus enabling better capture of rotational speed-independent discriminant features. According to the physical parameters and signals of the bearings, the fault characteristic frequencies of the inner and outer rings are approximately 5.43 $f_{r}$ and 3.57 $f_{r}$, respectively [36]. After the original signal has been transformed with the features, distinct fault features can be extracted from the angular domain spectral envelope. Simultaneously, prior knowledge of fault characteristics is employed in designing the anti-aliasing filter for angular domain resampling to eliminate the potential effects of rotational speed and bearing geometry properties on the apparent waveform of the signal [12].

**Figure 9:**
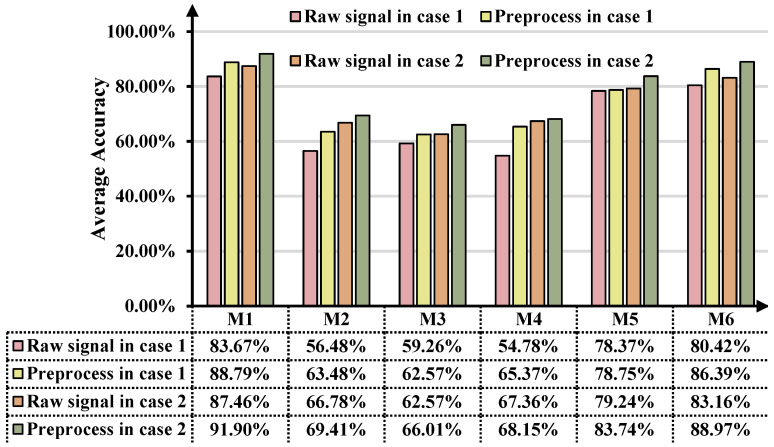
Comparison before and after angle domain resampling.

To demonstrate the necessity of eliminating the interference of time-varying rotational speed fluctuations, we conducted ablation experiments with or without preprocessing in two cases and calculated the average diagnostic accuracy of M1–M6 for all the DA scenarios. As shown in Figure 9, we compare the overall average accuracy differences between the five approaches in the two cases, revealing that angular resampling improves the cross-domain IFD method’s performance under time-varying rotational speed conditions. When speed-independent transferable features are used as the training inputs for ASY-WLB, the average diagnostic accuracy increases by 5.12% and 4.44%, respectively. Meanwhile, ASY-WLB is 2.40% and 2.93% higher than DCC’s second-highest accuracies of 86.39% and 88.97%. The average accuracies of M1–M6 are improved by 5.39% and 3.60%, respectively, and the performance improvement is especially obvious for M2 and M4.

### 3.4. Diagnostic Accuracy Analysis

**Figure 10:**
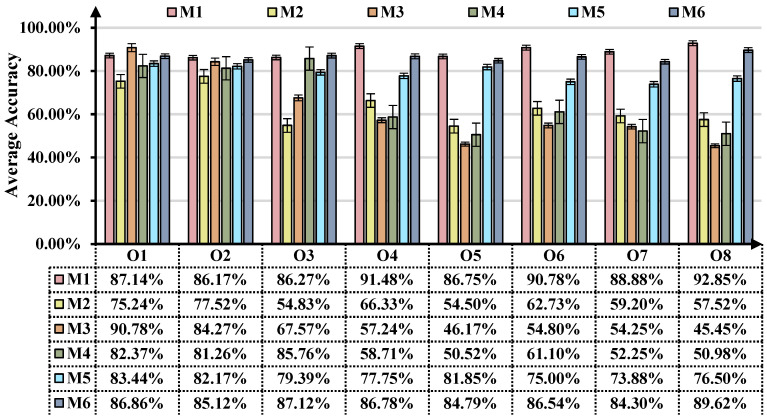
Comparison of overall accuracy of different tasks in case 1.

**Figure 11:**
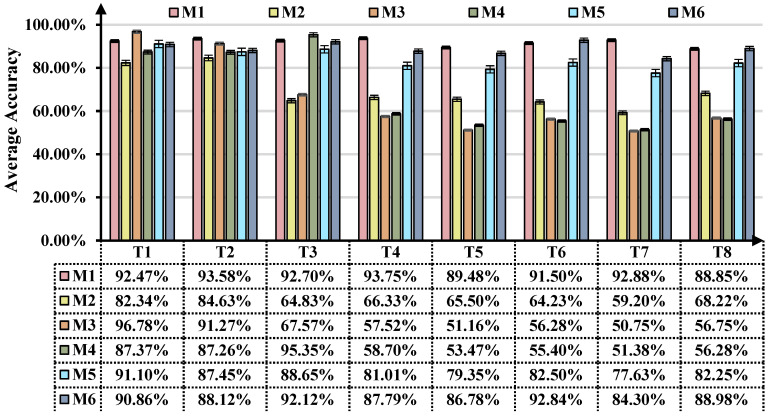
Comparison of overall accuracy of different tasks in case 2.

The statistical results and diagnostic result diagrams based on the two case studies can be shown in Figure 10 and Figure 11, respectively. The above experimental results reveal that the proposed ASY-WLB can attain the highest average diagnostic accuracy in the two case studies. It should be noted that the overall accuracy here denotes the average classification accuracy between the known and unknown classes in the target domain. In the two case studies, M2 failed to account for domain adaptation issues, resulting in the lowest diagnosis accuracy in all the UDA scenarios. M3 can achieve cross-domain IFD under domain label symmetry conditions by domain adversarial training and even achieve good results in PUDA scenarios. However, when unknown classes from the source domain emerge in the target domain, M3 is rendered helpless. Unlike M3, M4 maps the feature space of the source domain to the target domain and designs a strategy for handling outliers in the target domain. Therefore, it has obvious performance advantages in OSUDA scenarios compared to M2 and M3. However, M4 does not consider the outliers in the source domain relative to the target domain and can only force the knowledge of the shared classes to match the private classes, such that the diagnostic performance of M4 in the UniUDA scenarios is severely degraded.

The results for tasks O4–O8 and T4–T8 reveal that the diagnostic test accuracy of ASY-WLB in UniUDA scenarios is significantly higher than other methods. Meanwhile, M3 and M4 perform even worse than M2 when private classes exist in both the source and target domains. This could be owing to private class samples from different distributions being misaligned during the DA process, resulting in learning bias in the IFD model. It should be pointed out that, based on the results of task (T4, T5) and task (O4, O5), the classification accuracy increases as the number of private classes in the source and target domains decreases.

M1 embeds a label-positioning information compensation mechanism and WCDD loss in the training process. Compared to M5 or M6, on the one hand, it enables different domains to realize sample-wise alignment learning and class-wise alignment learning. On the other hand, M1 allows the source and target domains to aggregate the same classes of samples as much as possible in the feature space and amplify the distances of the samples of different classes to achieve sparseness of class decision boundaries. These reasons also give ASY-WLB a distinct advantage in performing cross-domain IFD with either symmetric or asymmetric domain-labeling space.

**Figure 12:**
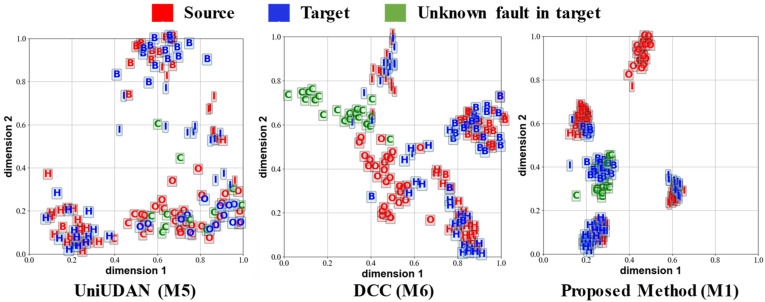
Output visualization of task O6 in case 1. H: healthy; I: inner-ring fault; O: outer-ring fault; B: rolling element fault; C: compound fault.

**Figure 13:**
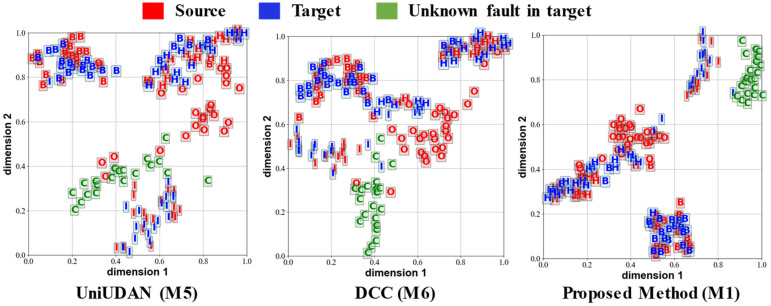
Output visualization of task O8 in case 1. H: healthy; I: inner-ring fault; O: outer-ring fault; B: rolling element fault; C: compound fault.

**Figure 14:**
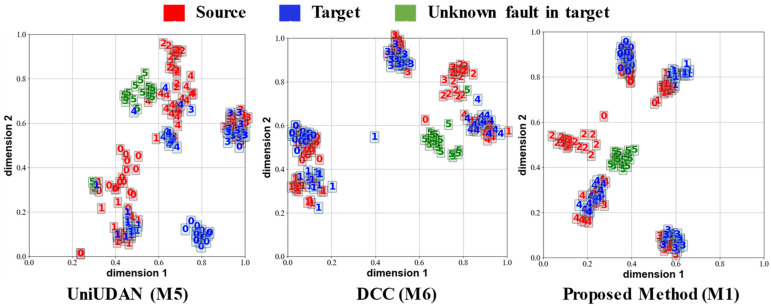
Output visualization of task T4 in case 2. 0: Healthy; 1: Compound fault C; 2: Inner defect I; 3: Planetary gear wear W; 4: Planetary gear crack; 5: planetary gear pitting.

**Figure 15:**
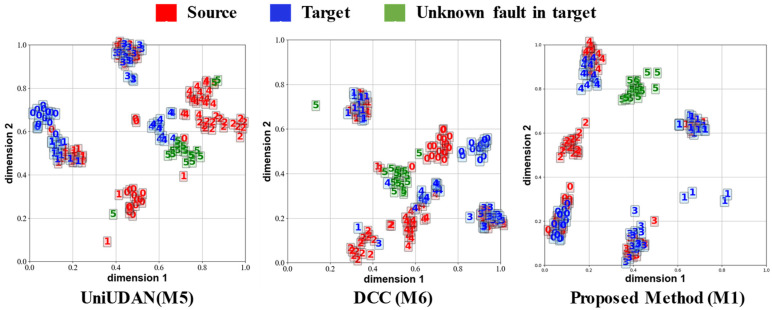
Output visualization of task T7 in case 2. 0: Healthy; 1: Compound fault C; 2: Inner defect I; 3: Planetary gear wear W; 4: Planetary gear crack; 5: planetary gear pitting.

Figure 12, Figure 13, Figure 14 and Figure 15 visualize the output features of certain UniUDA tasks in the two case studies using the t-Distributed Stochastic Neighbor Embedding (t-SNE) algorithm. We chose the methods M1, M5, and M6, which have the relatively best diagnostic performances, for comparison. The trend of rotational speed change in the source domain of Task O6 is singular, while the trend of rotational speed change in the target domain fluctuates substantially. The private class of the source domain is the outer-ring fault, while the unknown class of the target domain is compound faults. As shown in Figure 12, M5 can only align the distribution feature of healthy samples and rolling element fault samples in task O6. M6 accomplishes some degree of known class alignment and unknown class separation, but there is still a significant feature distribution coupling of decision boundaries for different classes to M1. Rotational speed changes in both the source and target domains of task O8 fluctuate sharply, but with the opposite trends (acceleration followed by deceleration in the source domain and deceleration followed by acceleration in the target domain). In this task, samples in both the source and target domains contain data under acceleration and deceleration conditions, so the samples in different domains are diverse and similar. As shown in Figure 13, although there is a small amount of confusion between the source domain private class features and the health class features in the output of the M1 method, our proposed method M1 still represents the most favorable level. M1 increases the distribution distance between classes, enhances the accuracy of recognizing unknown classes in the target domain, and summarizes the obvious clustering center and decision boundary.

Case study 2 utilized multiple channels of input data and contained more information per unit sample compared to case study 1. Also, a portion of the data in case study 2 was obtained under uniform speed conditions, making the diagnostic task in case study 2 less difficult. Figure 14 and Figure 15 visualize the output features for task T4 and task T8, respectively. The different classes of the sample features are better separated, and the source and target domains are better aligned compared to case study 1. Overall, compared with the most classic and advanced methods, the proposed ASY-WLB is still advantageous in different DA scenarios.

### 3.5. Parameter Sensitivity Analysis

**Figure 16:**
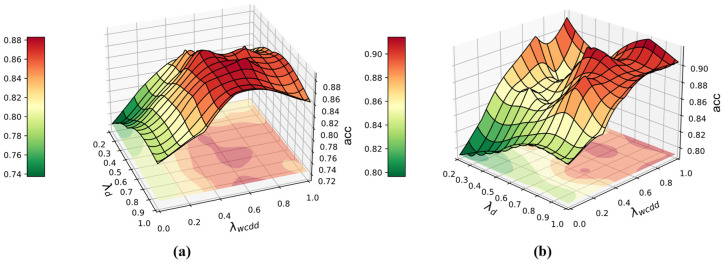
ASY-WLB with different parameter settings. (**a**) Task O7 in case 1 (**b**); task T6 in case 2.

In this section, we discuss the trade-off parameters $\lambda_{d}$ and $\lambda_{wcdd}$ in two case studies. We search for the optimal combination of these two trade-off parameters in task O7 and task T6, respectively. $\lambda_{d}$ determines the feature alignment capability of ASY-WLB for the source and target domains. $\lambda_{wcdd}$ determines the weight of the WCDD loss in the training process. When b is too small, we may obtain similar output distributions as methods M5 and M6, and sparsity around the class decision boundary is not guaranteed. When $\lambda_{wcdd}$ is too large, the balanced learning of intra-class compactness and inter-class separation interferes with domain adversarial training, as well as the label-positioning information compensation mechanism’s confidence discrimination for pseudo-labels in the target domain. Therefore, we need to search for the optimal combination of the two trade-off parameters $\lambda_{d}$ and $\lambda_{wcdd}$. We use the grid search method for $\lambda_{d}$ and $\lambda_{wcdd}$ in Task O7 and Task T6, respectively. For the blind region between the search nodes, we perform interpolation and smoothing and show the results in Figure 16.

## 4. Conclusions

In this paper, a cross-domain IFD method based on ASY-WLB is proposed, aiming at solving the asymmetry problem of domain label space under time-varying rotational speed conditions. On the one hand, we address the interference of the source domain’s private classes in the training process in UniUDA scenarios. On the other hand, we achieve feature separation and identification of unknown faults in the target domain. The conclusion of this paper is summarized as follows. (1) By combining physical a priori information and angular resampling, we can extract speed-independent transferable features under time-varying rotational speeds. (2) The label-positioning information compensation mechanism allows ASY-WLB to meet both instance-level and class-level alignment in non-adversarial learning, enhancing ASY-WLB’s perception of transferable features. (3) We propose a WCDD loss that realizes intra-class compactness and inter-class separation. Based on the above innovations, we demonstrate the applicability and superiority of ASY-WLB in a variety of cross-domain IFD scenarios by comparing it with the advanced methods.

Our methods have achieved significant results in solving the asymmetry problem of the domain label space under time-varying rotational speed conditions. However, our method also has some limitations. For example, the current method may still face challenges in handling extremely complex fault patterns or in scenarios with highly imbalanced data distributions. In future work, we plan to further improve the robustness and adaptability of the method by incorporating more advanced data-processing techniques and model optimization strategies. This could involve exploring more sophisticated feature extraction methods, developing more effective loss functions, or integrating additional prior knowledge into the model.

## Figures and Tables

**Table 1:** Asymmetric cross-domain IFD task information of case 1.

Task	Source Class (Inconsistent Speed Fluctuation of A, B, C, D)	Source Speed Varying Conditions	Target Class (Inconsistent Speed Fluctuation of A, B, C, D)	Target Speed Varying Conditions	DA Scenario
O1	HA, IA, OA, BA, CA	IS	HB, IB, OB, BB, CB	DS	CUDA
O2	HA, IA, OA, BA, CA	IS	HB, IB, OB, BB	DS	PUDA
O3	HA, IA, OA, BA	IS	HB, IB, OB, BB, CB	DS	OSUDA
O4	HA, OA, BA, CA	IS	HB, IB, OB, BB	DS	UniUDA
O5	HA, OA, BA	IS	HB, IB, BB, CB	DS	UniUDA
O6	HB, IB, OB, BB	DS	HC, IC, BC, CC	IS then DS	UniUDA
O7	HB, OB, BB	DS	HC, IC, CC	IS then DS	UniUDA
O8	HC, IC, OC, BC	IS then DS	HD, ID, BD, CD	DS then IS	UniUDA

**Table 2:** Asymmetric cross-domain IFD task information of case 2.

Task	Source Class (A: Load = 0.5 A; B: Load = 1 A)	Source Speed Varying Conditions	Target Class (A: Load = 0.5 A; B: Load = 1 A)	Target Speed Varying Conditions	DA Scenario
T1	HA1, CA1, IA1, WA1, KA1, PA1	IS	HA2, CA2, IA2, WA2, KA2, PA2	IS	CUDA
T2	HA1, CA1, IA1, WA1, KA1, PA1	IS	HA2, CA2, IA2, WA2, KA2	IS	PUDA
T3	HA1, CA1, WA1, KA1, PA1	IS	HA2, CA2, IA2, WA2, KA2, PA2	IS	OSUDA
T4	HA1, CA1, WA1, KA1, PA1	IS	HA2, CA2, IA2, WA2, KA2	IS	UniUDA
T5	HA1, CA1, KA1, PA1	IS	HA2, CA2, IA2, WA2	IS	UniUDA
T6	HB1, CB1, KB1, PB1	DS	HB2, CB2, IB2, WB2	DS	UniUDA
T7	HA1, CA1, IA1, WA1, KA1	IS	HB1, CB1, WB1, KB1, PB1	DS	UniUDA
T8	HA2, CA2, KA2, PA2	IS	HB2, CB2, IB2, WB2,	DS	UniUDA

## Data Availability

The data presented in case study 1 are openly available in https://doi.org/10.1016/j.dib.2018.11.019 [36]. Our data in case study 2 will soon be open for sharing.
